# Nuclear Nox4 interaction with prelamin A is associated with nuclear redox control of stem cell aging

**DOI:** 10.18632/aging.101599

**Published:** 2018-10-24

**Authors:** Francesca Casciaro, Francesca Beretti, Manuela Zavatti, James A. McCubrey, Stefano Ratti, Sandra Marmiroli, Matilde Y. Follo, Tullia Maraldi

**Affiliations:** 1 Department of Surgical, Medical, Dental and Morphological Sciences with Interest in Transplant, Oncology and Regenerative Medicine, University of Modena and Reggio Emilia, 41124 Modena, Italy; 2 Cellular Signalling Laboratory, Department of Biomedical and Neuromotor Sciences, University of Bologna, 40126 Bologna, Italy; 3 Department of Microbiology and Immunology, Brody School of Medicine at East Carolina University, Greenville, NC 27858, USA; 4 Cellular Signaling Unit, Department of Biomedical, Metabolic and Neural Sciences, University of Modena and Reggio Emilia, 41125 Modena, Italy

**Keywords:** AFSC, Nox4, nuclear ROS, prelamin, senescence

## Abstract

Mesenchymal stem cells have emerged as an important tool that can be used for tissue regeneration thanks to their easy preparation, differentiation potential and immunomodulatory activity. However, an extensive culture of stem cells *in vitro* prior to clinical use can lead to oxidative stress that can modulate different stem cells properties, such as self-renewal, proliferation, differentiation and senescence. The aim of this study was to investigate the aging process occurring during *in vitro* expansion of stem cells, obtained from amniotic fluids (AFSC) at similar gestational age.

The analysis of 21 AFSC samples allowed to classify them in groups with different levels of stemness properties. In summary, the expression of pluripotency genes and the proliferation rate were inversely correlated with the content of reactive oxygen species (ROS), DNA damage signs and the onset premature aging markers, including accumulation of prelamin A, the lamin A immature form. Interestingly, a specific source of ROS, the NADPH oxidase isoform 4 (Nox4), can localize into PML nuclear bodies (PML-NB), where it associates to prelamin A. Besides, Nox4 post translational modification, involved in PML-NB localization, is linked to its degradation pathway, as it is also for prelamin A, thus possibly modulating the premature aging phenotype occurrence.

## Introduction

Stem cells are implicated in preserving tissue homeostasis throughout life, by replacing damaged or lost cells. That is why an appropriate balance between self-renewal and differentiation is crucial for stem cell function during both early development and adult life. Recent studies suggest that this balance is partly regulated by reactive oxygen species (ROS) [1]. Indeed, physiological levels of ROS can have positive effects on the regulation of stem cell fate. In particular, oxidative stress is known to play an important role in modulating different stem cells properties, such as self-renewal, proliferation, differentiation and senescence [2].

The excessive generation of endogenous ROS and the imbalance between ROS and antioxidant systems, but also the culture of stem cells with various extracellular sources of ROS can lead to oxidative stress. This is the case of MSCs, whose low number requires *ex vivo* expansion prior to clinical use. At present, most of the expansion procedures of MSCs are performed under atmospheric O_2_ concentration (20% O_2_), which is approximately 4–10 times higher than the concentration of O_2_ in their natural niches [3]. This higher O_2_ concentration might cause environmental stress to the *in vitro* cultured MSCs, due to the increased ROS concentration. Recently, the review by Choi et al. [4] reported studies demonstrating that hypoxia has a greater ability to preserve the stemness of human Adipose SCs, (ASC) as indicated by the increased expression of stemness genes (e.g., NANOG, SOX-2, OCT4, and REX-1).

In general, oxidative stress due to high ROS levels impairs stem cell homeostasis and can induce DNA damage, cell cycle arrest and eventually a senescence phenotype [5]. Indeed, long-term ASC expansion at low O_2_ (5%) revoked in part the replicative senescence-associated alterations [6].

Senescence, defined as a series of cellular changes associated with aging, results from an impaired signal transduction program leading to irreversible arrest of cell growth and a distinct set of changes in the cellular phenotype [7,8]. Cellular senescence can be induced prematurely by various agents and stimuli [9] and the free-radical theory of aging postulates that the production of intracellular ROS is the major determinant of lifespan [8].

During physiological aging, minor forms of lipodystrophy are present and a slight prelamin A (PLA) accumulation is observed [10]. A-type lamins are nuclear proteins required for the structural and functional integrity of the nucleus. Lamin A is translated as a protein precursor that undergoes several maturation steps, including the addition of a C-terminal farnesyl residue, which is subsequently removed by proteolytic cleavage [11]. Defective physiological maturation of prelamin A is the main pathophysiological mechanism underlying several premature aging syndromes, including the Hutchinson-Gilford progeria syndrome (HGPS) [12].

It is becoming increasingly clearer that, next to its structural function and role in nuclear dynamics [13], the nuclear lamina also modulates intracellular redox homeostasis [14]. Various studies revealed not only that ROS levels are increased in laminopathy patient cells and during prelamin A accumulation, but also that this increase may be attributed to dysfunctional mitochondria [15–18]. Others studies observed that prelamin A accumulation in human MSCs gives rise to a premature aging phenotype that ultimately causes reduced functionality of these cells *in vivo* [19]. In fact, using a human *in vitro* model of LMNA-lipodystrophy, it has been shown that prelamin A accumulation causes defects in the differentiation of human MSCs to adipocytes [20]. Moreover, a number of studies indicates that the molecular mechanisms causing accelerated aging in progeric patients also occur in healthy cells of older population [21]. How fast and how healthy the aging occurs is also dependent on the individual specificity and its living habits.

The aim of this study was to investigate the aging process occurring in human MSCs during *in vitro* expansion. This process is certainly influenced by the oxidative stress exposure implied in extensive culture, but could also be donor dependent. The sample variability can be due both to the age of the donor and also to the individual characteristics. In order to restrict the age factor, stem cells obtained from amniotic fluids (AFSC), that is from foeti of similar gestational age, have been compared. The stemness properties, such as self-renewal and proliferation rate, have been correlated to the onset of senescence and premature aging markers. Then we investigated the relationship between a ROS producing enzyme and prelamin A accumulation into the nuclear foci of AFSC samples during the aging process. All in all, the results of this study may also explain some of the cellular changes occurring during normal aging, focusing on the role of stem cells subpopulation.

## RESULTS

### Characterization of hAFSCs cultures obtained from amniotic fluids of physiological pregnancy

21 amniotic fluids were collected at 16^th^-17^th^ week of pregnancy from healthy donors: foeti did not carry genetic abnormalities, pregnancies showed only in one case preeclampsia complication, but the Apgar score of all the new-borns was 9/10 and no one child needed assistance in neonatology. Women (average age 36.4) led healthy habits of life, except one smoker mother.

**Figure 1:**
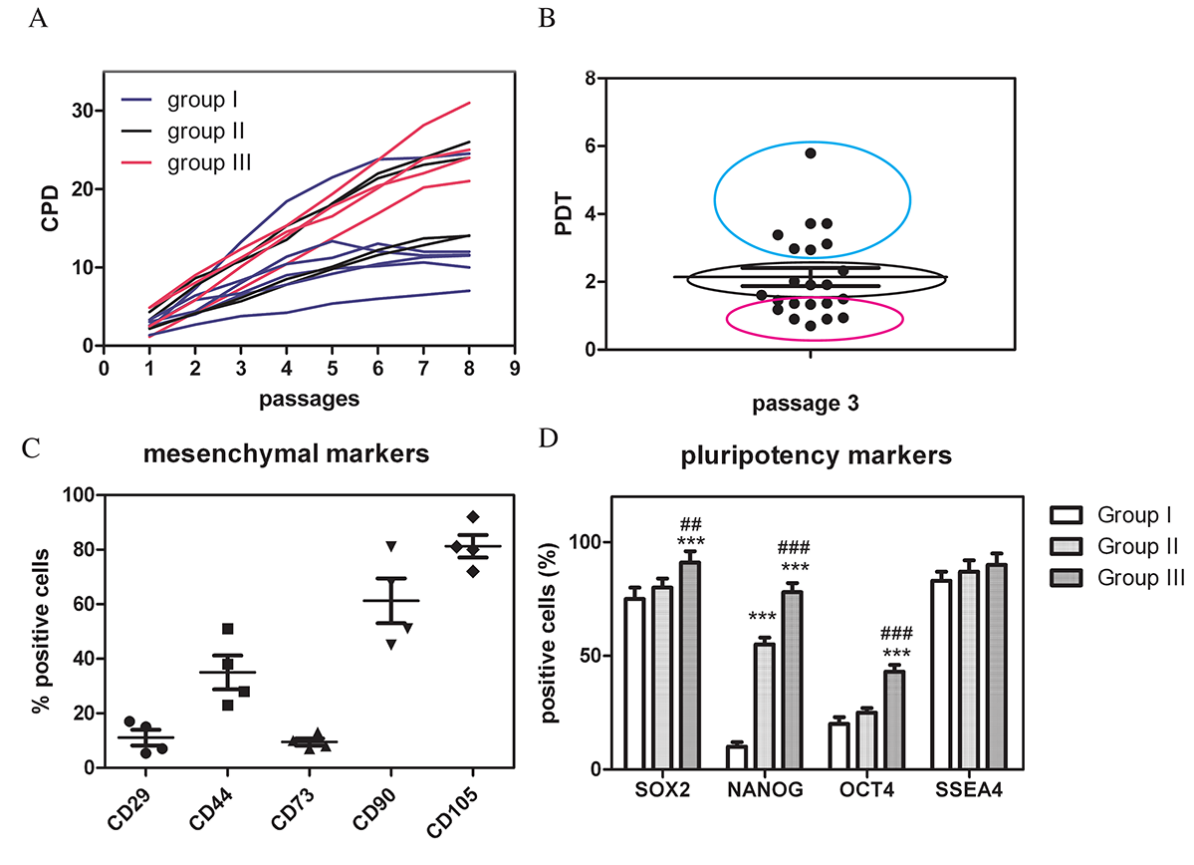
**hAFSCs derived from amniotic fluid of physiological pregnancy: comparison of the proliferation capability and stemness profile.** (**A**) The graph shows the cumulative population doubling (CPD) calculated up to the 7^th^ passage in culture for all the samples. (**B**) Population doubling time (PDT) values of all the samples, evaluated at the third passage in culture. (**C**) Quantitative analysis by flow cytometry of four representative samples for the expression of the surface markers: CD44, CD73, CD29, CD105, CD90. (**D**) Graph showing the cytofluorimetric analysis of Nanog, Oct4, Sox2, SSEA4 expression among the three groups. ***P < 0.0001; **P < 0.01 = significantly different from group I, ^###^P < 0.0001; ^##^P < 0.01 = significantly different from group II.

C-kit positive cells, selected from at least 1.5x10^6^ cells, were 1-4% of amniotic fluid cells. Analysis of cumulative population doubling (CPD) during passages (up to 7^th^ passage) established three groups of samples with different proliferation rate. The faster showed a trendline slope around 0.99 ± 0.01 *R^2^* and the slower was 0.96 ± 0.02 *R^2^*. A graph representative of three all the samples is shown in Figure 1A. Analysis of Population Doubling Time (PDT) at passage 3, therefore excluding the last part of the sigmoidal curve, confirmed that AFSC samples were distributed in three groups for the growth rate, as shown by coloured circles in Fig. 1B. Slower group showed a PDT value that doubled the faster one.

These data on AFSCs are consistent with the observation that different proliferative capability of MSCs cultures isolated from different donors exists [22,23]. The proliferation rate of AFSCs achieved its maximum level at passage 3 and, after passages 6-7, there was a decline in the efficiency of proliferation in all cell populations. Concurrently, also the analysis of flow cytometry was performed, to determine immunophenotype that still represents one of the major parameters for the characterization of MSCs cultures. As shown in Figure 1C, AFSCs from four representative samples were positive for mesenchymal markers, such as CD29, CD44, CD73, CD90, and CD105. Samples at early passages (not over the 4^th^ passage) maintained a quite similar level of cell surface markers CD29, CD44, CD73 and CD105, although CD90 seemed to be the more variable.

A potential marker of the senescent state of stem cells may be also a decreased expression of pluripotency transcription factors, such as Nanog, Sox2, SSEA4, and Oct4 [24]. Therefore, to confirm stem cell origin of AFSCs, we performed cytofluorimetric analysis of the main transcription factors from AF samples with slow (group I, n = 3), middle (group II, n=3) and high (group III, n = 3), proliferation (Fig.1D). Sox2 and SSEA4 were expressed at a quite similar level in all AFSC samples, while the expression of Oct4 and Nanog were much lower in slow samples (group I).

### Senescence-associated molecular changes in AFSCs cultures

**Figure 2:**
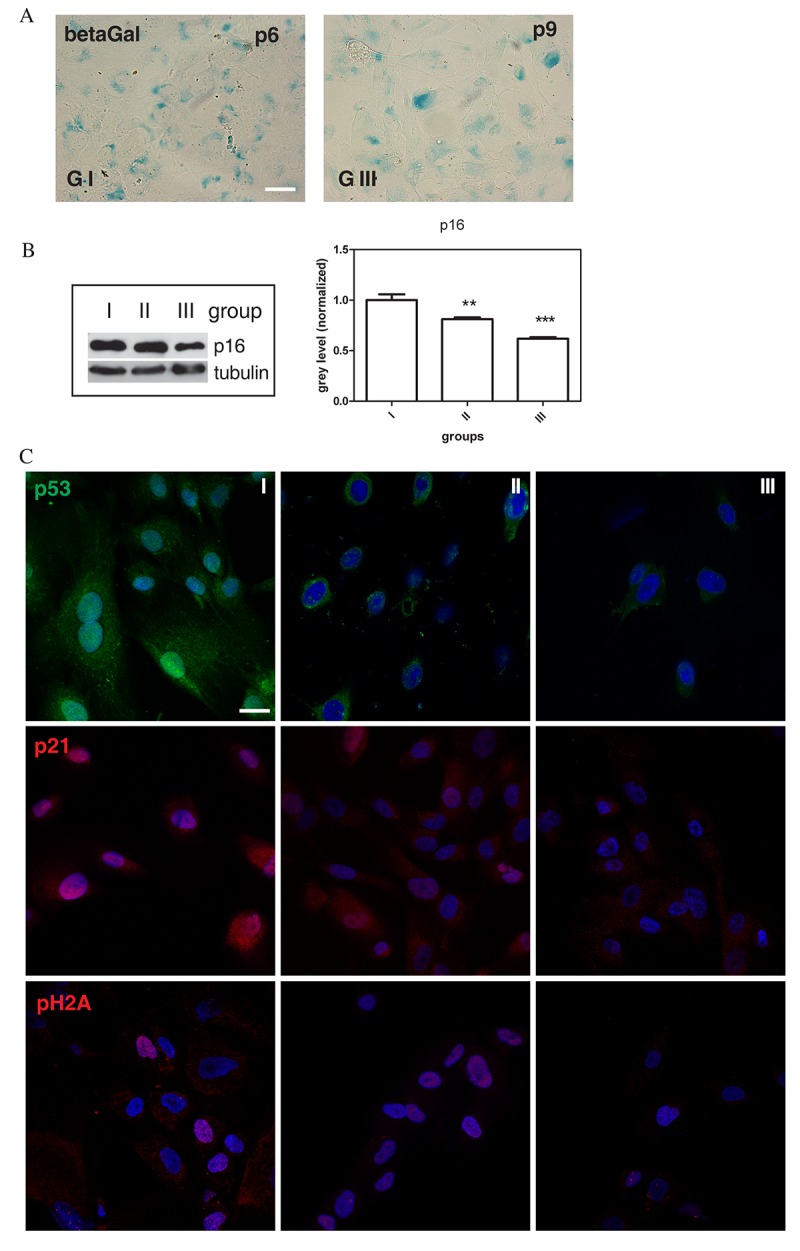
**Senescence-associated markers profile.** (**A**) Representative images of beta-galactosidase assay performed in group I (faster senescent cells) and group III (slower senescent cells) samples at passage 6 and 9, respectively. Senescent cells are blue stained. Scale bar=30 µm. (**B**) Western Blot analysis of total hAFSCs lysates revealed with anti-p16 antibody. Tubulin detection was performed as a loading control. The graph shows densitometric analysis of Western Blot experiment. Group I samples were set as 1. Data are representative of three independent experiments (***P < 0.0001; **P < 0.01). (**C**) Representative confocal images of group I, II and III samples double staining with DAPI (blue) and p53 (green), p21 (red), pH2A (red). Scale bar= 10 µM

Several biomarkers have been used for quantitative assessment of cell senescence, including senescence-associated 𝛽-galactosidase (SA-𝛽 gal) and the cyclin-dependent kinase inhibitors, p16INK4A and p21WAF1, that are involved in the control of growth arrest by two major tumour suppressor pathways, p16INK4A/pRb and p53/p21WAF1 [25,26]. As shown, during passaging of cell cultures derived from different AF donors, AFSCs cultures differently stop their growth in the course of cultivation. In fact, we observed that AFSCs senescence was accompanied by typical morphological changes and cells became enlarged and flattened, with enhanced SA-𝛽-gal activity (Figure 2A), while stem cell marker expression decreased, as reported for Oct4, as a representative marker, in Supplementary Figure S1. These senescence-associated changes occurred later in AFSCs samples that showed a relatively faster level of proliferation. Indeed, at early passages a slight more intense positivity for SA-𝛽-gal activity is more evident for group I than group III (Supplementary Figure S2), while the same amount (about 70%) of SA-𝛽-gal-positive cells was detected in groups I and III at p6 and p9 (denoted as late passage), respectively.

To gain insight into the molecular characteristics of AFSCs senescence, we analysed protein level profiles of classical senescence-associated markers, p16INK4A (p16), p21WAF1 (p21), p53, and pH2A.X as DNA damage index, in all groups of cell cultures at late passage. As demonstrated by Western Blot analysis, AFSCs cultures of group I showed higher p16 expression, as compared to group III (Figure 2B), so the level of p16 expression seemed to be inversely correlated with the proliferation capability. Moreover, we observed by confocal images more positivity for p21 and p53 expression in faster senescent cells (group I) than in group III, both in samples at late passage and at early passage, as shown in Figure 2C and S2. The analysis of the expression of pH2A.X showed a higher number of positive cells in group I, suggesting a correlation with expression levels of p16, p53/p21.

### Difference in nuclear redox state of AFSC groups

**Figure 3:**
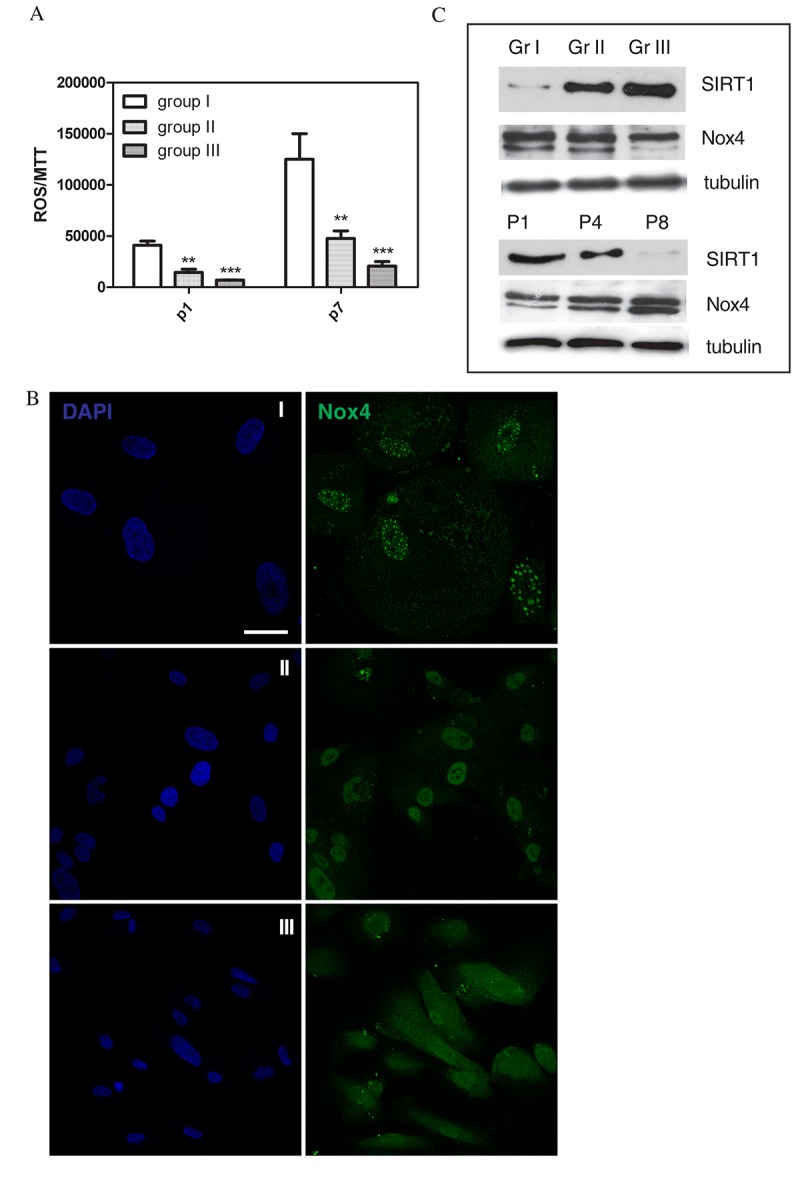
**ROS levels and ROS modulating proteins in AFSC groups.** (**A**) Representative graph showing fluorescence, obtained with ROS probe (DCFH-DA), normalized to MTT viability values of hAFSCs samples at 1^st^ and 7^th^ culture passage. ***P < 0.0001; **P < 0.01 significantly different from group III. (**B**) Representative images showing DAPI (blue) and Nox4 (green) signals of the three different hAFSCs groups. Scale bar= 10 µm. (**C**) Representative images of Western blot analysis of AFSC samples, group I, II and III (Gr I-Gr III, faster to slower senescent cells) at passage 4, or group II at passage 1, 4 and 8 (P1-P8), revealed with anti-Sirt1 and anti-Nox4 antibody. Tubulin detection was performed as a loading control. Data are representative of three independent experiments.

Since oxidative stress can cause DNA damage, we tested total reactive oxygen species (ROS) levels in cultures at early (p1) and late passages (p7). The graph of Figure 3A shows that samples of group I (faster senescent cells) have a content of ROS 10 times higher than group II, both at the first and at the seventh passage.

Moreover, we have previously demonstrated that a ROS producing enzyme, the isoform 4 of NADPH oxidase (Nox4), localizes in a spot distribution into the nuclei of AFSC, depending on their cell cycle state [27]. Here we can confirm this observation, thanks to a larger cohort of samples better characterized for clinical aspects and stem cell features. Indeed, the higher Nox4 nuclear localization in group I is shown in confocal images of Fig. 3B. Interestingly, the nucleoplasmic Nox4 is, in some images, not only in a spot-like but also in a ring-like distribution. Western blot analysis of total lysates (Fig.3C) indicates the increase, during passaging of group II AFSC, of a double band at 74-72 kDa. Moreover, the highest intensity of 72 kDa band is detectable in group I (faster senescent cells).

Sirtuins are a class of NAD+-dependent protein deacylases with important implications in aging [28]. Humans have 7 sirtuins (SIRT1–7) with distinct subcellular localizations and functions. The regulatory role of Sirtuin 1 (SIRT1) is predominantly linked to p53 activity. Interestingly, its activation in aged cells protects the cells from p53-dependent apoptosis or senescence [29]. It was found that oxidative stress regulates these sequences and promotes shuttling of SIRT1 [30,31]. Here we found that samples of group I have an expression of SIRT1 much lower than group III, and the effect of culture passaging is a decrease of SIRT1 expression, as expected (Fig. 3C).

### Nox4 and prelamin A interaction in AFSC groups

**Figure 4:**
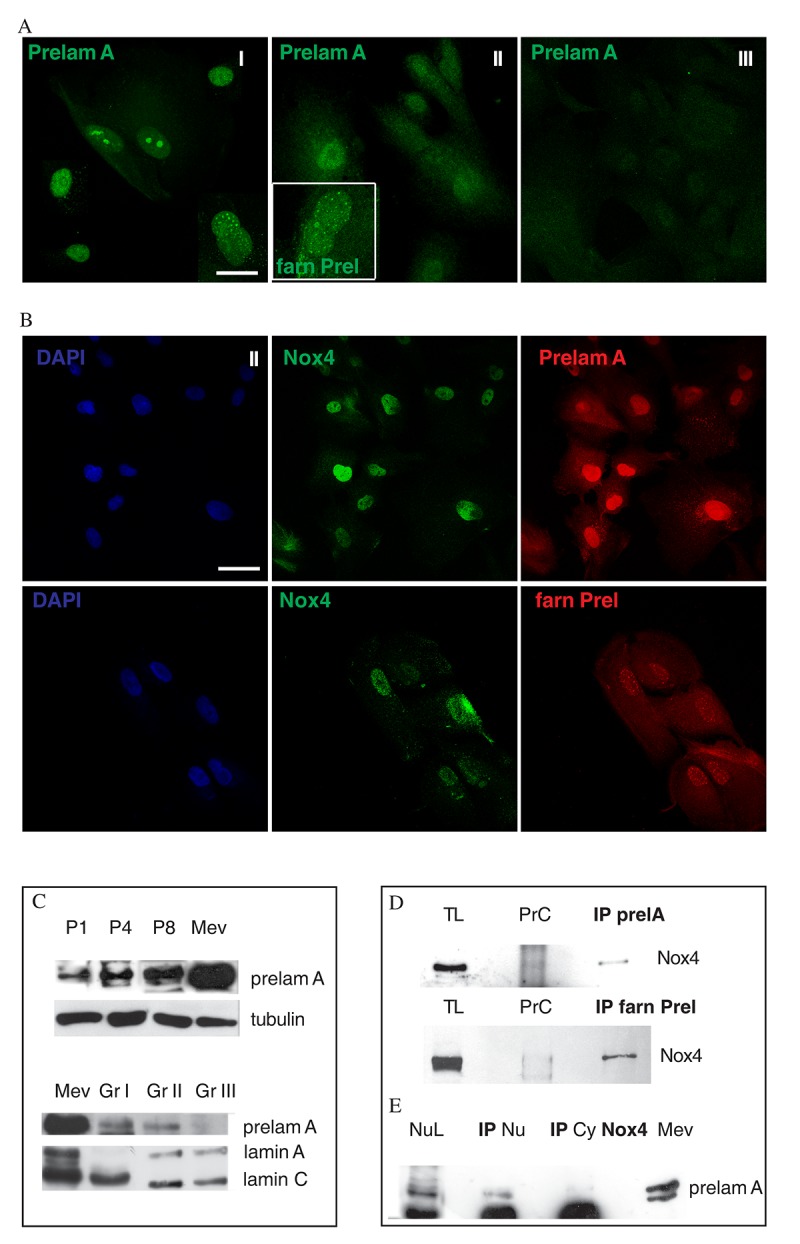
**Nox4 interaction with prelamin in nuclei of AFSC.** (**A**) Representative images of AFSC group I, II and III (faster to slower senescent cells) showing green signals of Prelamin A (Prelam A), and, in doubled magnification image in white square of group II, farnesylated Prelamin A (farn Prel). Scale bar= 10 µm. (**B**) Representative images of AFSC group II labelled with DAPI (blue), Nox4 (green) and Prelamin A (red) or farnesylated Prelamin A (red). Scale bar=10 µm. (**C**) Western Blot analysis revealed with anti-Prelamin A or the specific anti-Lamin A/C antibodies of total hAFSCs lysates of group II at passage 1, 4 and 8 and of group I, II and III at passage 4. Tubulin detection was performed as a loading control. Mevinolin (Mev) treatment was performed to show a prelamin A positive control. Data are representative of three independent experiments. (**D**) Western blot analysis of total lysate (TL) and immunoprecipitation (IP) experiment of TL with Prelamin A or farnesylated Prelamin A antibody then revealed with anti-Nox4. Preclearing samples were also loaded, in order to exclude non-specific signals. Data are representative of three independent experiments.(**E**) Western blot analysis of nuclear lysate (NL) and immunoprecipitation experiment of Nuclear lysate (IP Nu) and Cytoplasmic lysate (IP Cy) with Nox4 antibody then revealed with anti-Prelamin A. Mevinolin (Mev) treatment was performed to show a prelamin A positive control. Data are representative of three independent experiments.

Preliminary mass spectrometry analysis of proteins co-immunoprecipitating with Nox4 extracted from isolated nuclei hinted at the interaction of Nox4 with lamin A proteins (Supplementary Figure S3). However, since nuclear Nox4 staining does not overlap neither with nuclear envelop nor with nucleoli, we wondered if this interaction could involve an immature form of lamin A, such as prelamin A. Immunostaining of prelamin A and farnesylated prelamin A in AFSC revealed an extensive presence of punctate dots and fibrous aggregates at later passages in group II (Fig. 4A), but also at early passage in group I (faster senescent cells). Interestingly, some cells from group I samples showed a drop-like staining, likely localized into nucleoli.

Double staining for prelamin A and pH2A.X revealed that the simultaneously labeling in the same cell scarcely occurs, as shown in Supplementary Figure S4.

Conversely, double staining for Nox4/prelamin A and Nox4/farnesylated prelamin A showed a partial colocalizations of these two proteins in the nuclear spots (Fig. 4B).

This prelamin A accumulation, during passaging, was confirmed also by Western blot analysis (Fig. 4C), suggesting a positive correlation between prelamin A expression and cell aging, parallel to Nox4 increase. Indeed, Western blot of Figure 4C probed with anti-prelamin A demonstrates the presence of a higher band (74 kDa), that is more expressed in samples from group I (faster senescent cells), although at lower intensity as compared to the positive control (cells treated with mevinolin). Moreover, the band corresponding to mature lamin A decreased: this effect is consistent with an accumulation of its immature form, prelamin A. The accumulation of nuclear Nox4 and prelamin A can be reduced by the ROS decrease, by treating cell culture with natural antioxidants, as shown in Figure S5. These two phytochemicals, sulforaphane (SF) and epigallocatechin gallate (EGCG), were chosen since they possess different chemo-physical properties (EGCG is more hydrophilic than SF) and because they could modulate various cell pathways.

Moreover, this increase is negatively correlated to the adipogenic differentiation capability of AFSC: Figure S6 shows that group II, that presents a medium stemness profile but accumulates prelamin A, after exposure to adipogenic medium for 3 weeks, expressed a significantly lower level of the adipogenic marker PPARγ, compared to group III.

In Fig. 4D AFSC total cell extracts immunoprecipitated with an anti-prelamin A or farnesylated prelamin A antibody and then immunoblotted with anti-Nox4 are shown. We detected high molecular weight (74 kDa) band indicative of Nox4 interaction with both the immature forms of lamin A.

This interaction was confirmed by immunoprecipitation of nuclear lysates with anti-Nox4 antibody, detected for anti-prelamin A, as shown in Figure 4E.

### Nox4-prelamin A interaction: what is the meaning?

It has been reported that SIRT1 is recruited by promyelocytic leukemia (PML) protein to nuclear bodies (PML-NBs) and SIRT1 can rescue mouse embryonic fibroblasts from PML-induced premature senescence [32]. Given the involvement of PML-NBs in aging and in protein sequestration/degradation and the similarity between their structure and that of Nox4/prelamin A intranuclear aggregates, we hypothesized that these compartments might be involved in their nuclear accumulation.

**Figure 5:**
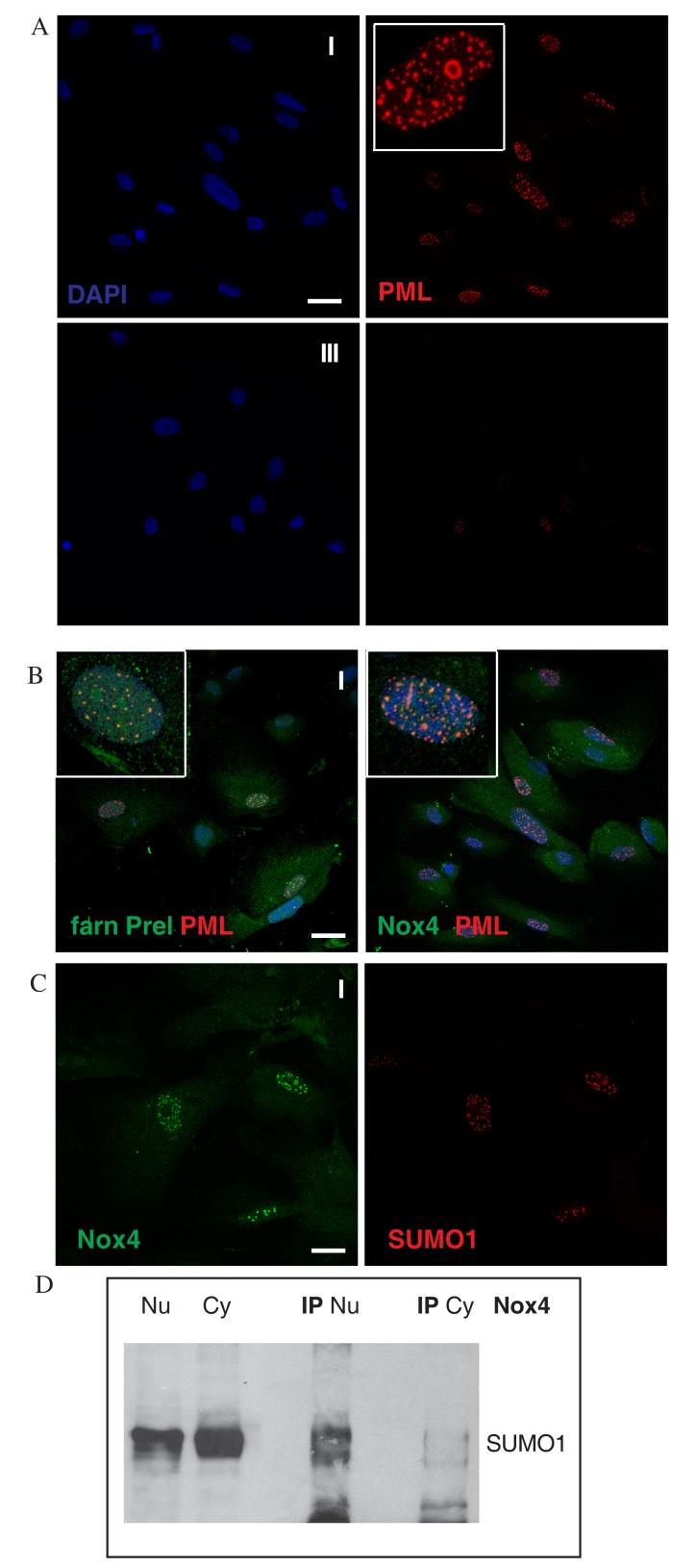
**Nox4 post-translational modification in nuclei of AFSC.** (**A**) Representative images showing DAPI (blue) and PML (red) of AFSC of group I and III. Tripled magnification image is present in white square of group I. (**B**) Representative images of AFSC group I (faster senescent cells) showing superimposing of DAPI (blue), farnesylated prelamin A (green) or Nox-4 (green) and PML (red). Tripled magnification image is present in white square of both. Scale bar= 10 µm. (**C**) Representative confocal image of AFSC group I samples labelled with Nox4 (green) and SUMO1 (red). Scale bar= 10 µM. (**D**) Western blot analysis of nuclear lysate (Nu), cytoplasmic lysate (Cy) and immunoprecipitation experiment of Nu and Cy with Nox4 antibody then revealed with anti-SUMO1. Data are representative of three independent experiments.

We first explored the localization of PML protein in the different AFSC groups. PML-NBs morphology and number were low in patients of group III (slower senescent cells), with PML-NBs appearing as classical punctate structures (Fig.5A). In group I (faster senescent cells), the dimension of classical PML-NBs increased and the number of aberrant ring-like and thread-like PML-NBs appeared (Fig.5A).

We then checked whether prelamin A colocalized with these structures, and we could observe that this was the case (Fig.5B). Double immunostaining with anti-PML and anti-prelamin A antibodies on AFSC group I using confocal microscopy showed that part of intranuclear farnesylated prelamin A content colocalized with the PML bodies, seeming to be sequestered inside them in some regions. A similar result was obtained for Nox4 and PML double staining.

PML protein is degraded through a SUMO-triggered RNF4/ubiquitin-mediated pathway, and most PML-associated proteins undergo SUMO-conjugation [33]. To investigate post translational modification of nuclear Nox4, we carried out immunofluorescence experiments, using Nox4 and SUMO1 antibodies on AFSC from group I (faster senescent cells). As shown in Fig.5C, Nox4 and SUMO1 staining colocalized into PML-like intranuclear foci.

Western blot using SUMO1 antibody in Nox4 immunoprecipitated nuclear or cytoplasm lysates revealed that nuclear Nox4 is directly linked to SUMO1 or through another sumoylated protein with 74 kDa molecular weight, such as prelamin A (Fig. 5D), corroborating Nox4 localization within PML-NBs.

**Figure 6:**
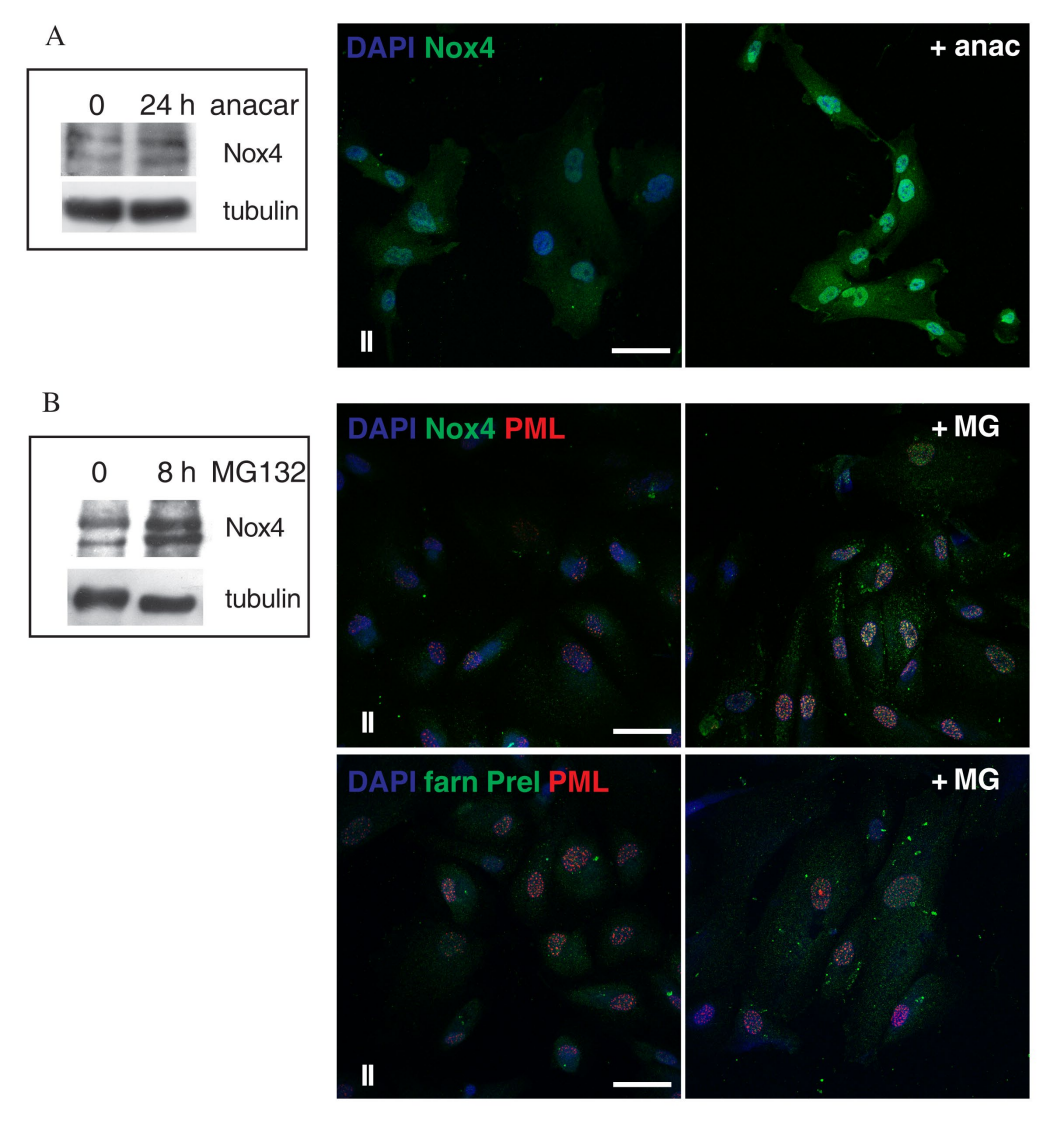
**Effect of Anacardic Acid and MG132 treatment on Nox4 expression.** (**A**) Representative image of Western blot analysis of AFSCs group II samples (intermediate senescent cells) used as control (0) or treated with anacardic acid for 24 h and then revealed with anti-Nox4. Tubulin detection was performed as a loading control. Data are representative of three independent experiments. On the right, representative images showing superimposing between DAPI (blue) and Nox-4 (green) of control or anacardic acid treated samples. Scale bar= 10 µm. (**B**) Representative image of Western blot analysis of AFSCs group II samples used as control (0) or treated with MG132 for 8 h and then revealed with anti-Nox4. Tubulin detection was performed as a loading control. Data are representative of three independent experiments. On the right, representative images showing superimposing among DAPI (blue), Nox-4 (green) or farnesylated Prelamin (green) and PML (red) of control or MG132 treated samples. Scale bar= 20 µm.

To better determine the effect of Nox4 sumoylation, we treated AFSC of group II, since it shows intermediate features, with a membrane permeable sumoylation inhibitor (anacardic acid) [34]. Data shown in Figure 6A, by Western blot and immunofluorescence analyses, highlighted that SUMO inhibition caused an increase of Nox4 presence, both nuclear and cytoplasmic, suggesting that the inhibition of general sumoylation causes a block in the degradation pathway of Nox4.

To determine whether proteasome inhibitors may influence the levels of nuclear Nox4, we performed treatment with MG132, an inhibitor of the proteasome pathway, and investigated Nox4 expression by Western blot and localization by immunofluorescence. Figure 6B indicates that MG132 induced an increase in Nox4 levels both in the cytoplasm but also into the PML nuclear foci. Since it has been demonstrated [35] that the effect of MG132 in Hutchinson-Gilford progeria syndrome (HGPS) fibroblasts unexpectedly resulted in a decrease of progerin staining intensity, instead of increasing progerin levels, we carried out immunofluorescence experiments for farnesylated prelamin A. As shown in Fig.6B, 8-h treatment with MG132 induced the formation of large farnesylated prelamin A and PML intranuclear foci, not a decrease.

## DISCUSSION

ROS have a role in DNA damage accumulation in human stem cells and have a major impact on stem cell aging. Indeed, our results show that there are differences in the stemness profile of AFSCs during *in vitro* expansion, due to donor variability. One third of AFSC samples showed a low proliferation rate, linked to poor expression level of pluripotency markers and faster senescence occurrence, as well as higher total ROS production. Interestingly, these phenomena were accompanied to the accumulation in the nucleoplasm of lamin A precursors, unexpected effect since these are stem cells of foetal age. At the same time, the presence of Nox4, a ROS enzymatic source, increased in the sumoylated form into the nuclei localizing in PML bodies and interacting with immature lamin A. This localization is a way to sequester and favour proteasome degradation, with the possible protecting meaning to modulate ROS content, limit DNA damage and induce senescence.

Our study was based on the statement that *in vivo* stem cells reside within a hypoxic niche that preserves MSC progenitor properties [36], while *in vitro* culture of stem cells is routinely performed at atmospheric oxygen tension that leads to abnormal production of ROS. The excess of intracellular ROS deeply impacts on stem cell functionality, particularly proliferation and differentiation [37], thus mimicking and shortening the aging process. We hypothesized, however, that an intrinsic ROS susceptibility, depending on individual variability, is an important modulator in stem cell power, both *in vitro* and *in vivo*. Indeed, here we confirmed that stem cells of the same foetal age, taken from healthy pregnancies, show marked differences in the proliferation rate and in the timing of senescence occurrence since the first passage in culture. In fact, slower samples express low levels of stemness markers, since the earliest passages in culture. However, we did not observe significant differences in mesenchymal marker expression, and this may be due to our analyses, that were all conducted at the same early passage. Our findings are consistent with the study of Bertolo et al. [38] where adult MSC isolated from donors of the same age showed distinctive behaviour in culture, demonstrating the importance of mitochondrial function in MSC in vitro, and that cells with low oxidative status levels are better candidates for cell-based therapies. Collectively, our data suggest a picture of donor-dependent heterogeneity in stem cells efficacy, that not only points the attention to the need to attentively screen stem cells samples prior to undertake regenerative therapy approaches, but it could also be a mirror of an intrinsic and individual inclination to a premature aging progression.

The response of stem cells to oxidative stress is not well understood, but it has been related to increased double-stranded DNA breaks, faster telomere shortening for MSCs cultured in ambient condition [39] and prelamin A accumulation, which may be due to mitochondria derived-ROS that give rise to a premature aging phenotype [40]. Here we showed that low oxidative status levels, obtained with antioxidant treatment, prevented nuclear prelamin A accumulation. Indeed, alteration in the nuclear profile and in chromatin organization, which are particularly impressive in systemic laminopathies, whose cells undergo premature senescence, are mainly due to accumulation of unprocessed prelamin A [17]. In fact, depletion of ZMPSTE24, the farnesylating enzyme of prelamin A, can trigger a senescence pathway associated with moderately increased ROS levels and transient Δψ_m_ depolarization [41]. These considerations are consistent with our data in the AFSC samples with a poor growth and stemness profile (group I), showing prelamin A/farnesylated prelamin A accumulation and a higher intracellular ROS content. The expression of prelamin A in cells of so young origin is still surprising but our data, comparing adipogenic differentiation capability of AFSC of different groups, are consistent with another paper reporting that, in an *in vitro* model of LMNA-lipodystrophy, prelamin A accumulation causes defects in the differentiation of human MSCs to adipocytes [21]. We also noticed by immunofluorescence analysis that the cells positive for prelamin A rarely presented DNA damage markers, data that could suggest a protective role for prelamin A accumulation.

Moreover, it has been reported that the nuclear lamina itself can also modulate intracellular redox homeostasis [14]. We previously reported that the nuclear localization of a ROS source, Nox4, is peculiar of some AFSC samples, with a correlation with the cell cycle state [27], but here we observed that Nox4 expression, during passaging or comparing cells of group III, II and I (slower to faster senescent cells), moves from the cytoplasm to a nuclear spot like distribution, and that this phenomenon occurs in AFSC already expressing farnesylated prelamin A, driving to a senescent scenario. Interestingly, we noticed that some AFSC samples of group III (slower senescent cells) express farnesylated prelamin A, but do not show nuclear Nox4 distribution, suggesting that the modification in lamin A processing is an upstream event, as compared to Nox4 accumulation into the nuclei. Taking into account this consideration, we can look at the interaction with Nox4 inside the nucleoplasm as a response to the general redox imbalance occurring in these cells. In fact, a ROS decrease, due to antioxidant treatment, caused a decrease on prelamin and Nox4 nuclear signals, hinting at a role for high ROS as essential drivers of cellular aging. Moreover, the colocalization between these proteins is particularly evident into PML nuclear bodies. Indeed, promyelocytic leukemia nuclear bodies (PML-NBs), discrete nuclear speckles tightly associated with the nuclear matrix and implicated in cellular senescence [42], have a nuclear arrangement that is influenced by A-type lamin deficiency [43]. Also, our data show an increase in PML-NBs dimensions and abnormal features in AFSC of group I (faster senescent cells), that contain high prelamin A levels and low SIRT1 expression, compared to group III. Interestingly, SIRT1 has been reported to be recruited by PML to PML-NBs, where it co-localises with p53, through inhibition of proapoptotic activity of p53 [32]. In this way, the regulation of SIRT1-p53 axis can modulate stem cell fate. Indeed, p53 can also regulate miR-145 expression that is negatively associated with many genes involved in cell metabolism [44].

PML-NBs regulate posttranslational modifications of partner proteins like sumoylation, ubiquitination, but also phosphorylation or acetylation. These modifications involve a wide variety of partners, leading to modulation of biological processes like transcription, apoptosis/senescence, DNA repair, and stem cell self-renewal.

SUMO is conjugated to target proteins on the side chain of lysine residues, creating a branched peptide and significantly changing the binding properties of the protein. SUMO has been implicated in multiple pathways, mostly as a regulator of protein interactions [45]. Interestingly, some reports have demonstrated sumoylation of lamin A at lysine 201 [46]. This sumoylation site is also present in prelamin A and could be involved in its translocation to PML-NBs, therefore we focused our attention only on the possible sumoylation of Nox4. It has been reported that, upon heat shock, SUMO1 is attached to another isoform of NADPH oxidase (Nox2), thus blocking its activity and consequently controlling redox status and protecting the cell from oxidative stress-induced death [47]. However, the analysis of Nox4 sequence highlighted the presence not only of sumoylation sites but also the presence of SUMO interaction motifs (SIM) close to them, that were very numerous.

Since conjugated SUMO may interact with the short motif SIM and most partner proteins associated with classical PML-NBs are sumoylated and many contain a SIM motif, SIM/SUMO interactions may also account for partner recruitment as well as sequestration [48]. In this context, here we demonstrated the presence co-immunoprecipitation of SUMO1 with nuclear Nox4, suggesting that SUMO modifications participate in Nox4 and prelamin interactions and cellular senescence, through modulation of oxidative stress responses.

PML-NBs have been proposed to function also as sequestration sites of misfolded proteins targeted for proteasomal degradation [42]. However, the effect on progerin protein and treatment with proteasome inhibitor was not obvious, as Harhouri et al. observed progerin decrease upon proteasome inhibition that suggested that progerin could be a substrate for autophagic degradation as a compensatory response [35]. That is why we tested the effect of the proteasome inhibitor MG132, on farnesylated prelamin A and Nox4. Our data, also the one with the sumoylation inhibitor, confirmed that PML-NB sequestration via sumoylation modification may have a role in the proteasome degradation of these interacting proteins, events that could create an elimination flow of the ROS source Nox4, concentrating this protein in nuclear domains where a response to redox stress could be triggered and avoiding DNA damage maintaining the cells in a senescence state.

In conclusion, these data demonstrate that, also in the younger and more plastic cells of our body, like AFSCs, differences in the capability to counteract oxidative stress exist among the individuals. Also, in the more sensitive cells protective mechanisms are activated, but these are signs of poor self-renewal properties for stem cells that are not efficient for therapeutic purpose.

## MATERIALS AND METHODS

### Amniotic fluid collection

The hAFSCs were obtained from 21 amniotic fluids collected from women (average age 36.4 ± SD 3.2) between the 16^th^ and 17^th^ week of gestation who underwent amniocentesis at Arcispedale S. Maria Nuova Hospital in Reggio Emilia. During the pre-amniocentesis interview, pregnant women were informed of the purpose of the study and of any risk related to. The informed consent was obtained, in accordance with the Italian law and the guidelines of the ethics committee (protocol 2015/0004362 dated 02.24.2015). Informed consent, as well as all documentation relating to the invasive procedure, were signed by the pregnant women and by an ob-gyn specialist before continuing the exam.

Supernumerary (unused) flask of AF cells cultured in the Laboratory of Genetics Arcispedale S. Maria Nuova for 2 weeks were trypsinized and treated to be freezed.

### Adult human tissue isolation and cell culture

Human amniotic fluid stem cells (hAFSCs) were isolated as previously described by De Coppi et al. 2007 [49]. Human amniocentesis cultures were harvested by trypsinization, and subjected to c-Kit immunoselection by MACS technology (Miltenyi Biotec, Germany). AFSC were subcultured routinely at 1:3 dilution and not allowed to expand beyond the 70% of confluence. AFSC were grown in culture medium (αMEM supplemented with 20% fetal bovine serum (FBS), 2 mM L-glutamine, 100 U/ml penicillin and 100 μg/ml streptomycin (all from EuroClone Spa, Italy) at 37°C 5% CO_2_.

### Adipogenic differentiation

Cells were incubated for 3 weeks in adipogenic induction medium (culture medium supplemented with 0.5 mM isobutylmethylxanthine, 1 mM dexamethasone, 10 mM insulin, 200 mM indomethacin, and 10% FBS). Medium was changed twice a week. Lipid-rich vacuoles within the cells were evaluated by Oil red O staining and AdipoRed assay (LONZA, Basel Switzerland), following the manufacturer’s instructions.

### ROS detection

To evaluate intracellular ROS levels, dichlorodihydro-fluorescein diacetate (DCFH-DA) assay was performed as previously described [50]. Cells were seeded in 96 well plate at density of 3000 cells/well, 6 replicates. Cell culture medium was removed and the 5 μM DCFH-DA was incubated in PBS glucose 5 mM for 30 min, 37°C and 5% CO_2_. The cell culture plate was read at 485 nm (excitation) and 535 nm (emission) using the Appliskan instrument (Thermo Fisher Scientific, Vantaa, Finland).

### Cell viability assay

Viable cells were evaluated by the MTT assay. Cells were incubated with 0.5 mg/mL MTT for 4 h at 37 °C, as previously reported [50]. At the end of the incubation, purple formazan salt crystals were dissolved by adding the solubilization solution (isopropanol, 0.1 M HCl). The absorption at 570 nm was measured on a multiwell plate reader (Appliskan, Thermo Fisher Scientific, Vantaa, Finland).

### Cell proliferation assays

The proliferation rates of hAFSCs were analysed as previously reported [51]. After reaching confluence, adherent cells, isolated as described above, were trypsinized and seeded in T25 cm^2^ flask at a density of 2x10^3^ cells/cm^2^, cultured for 3.5 days then detached, counted and seeded again at 2x10^3^ cells/cm^2^. Cell density was expressed as the mean of cells/cm^2^ ± SD. Cultures were performed until passage 8. The following formula was applied to all samples:

PD = [log_10_NH – log_10_NS]/ ÷ log_10_2

where PD is the population doubling, NS is the cell number at seeding (2 × 10^3^ cells/cm^2^) and NH is the cell number at harvest. To calculate the cumulative number of population doublings (CPD), the PD determined for each passage was then added to the CPD of the previous passage.

The population doubling time (PDT) was calculated in the phase of exponential growth by the following formula:

PDT log_10_(2) x ΔT/log_10_(NH)-log_10_(N1d)

where ΔT represents the time between the cell harvesting and seeding, NH represents the harvested cell number and N1d represents the cell number at day 1.

### Senescence assay

In order to evaluate the presence of senescent cells in AFSCs of samples, cells at passage 7-9 were seeded in 12-well plates and processed using a senescence β-Galactosidase staining kit (Cell Signaling, MA, USA), according to the manufacturer’s instructions.

### Flow cytometer immune-assay

AFSCs at first passage were sub-cultured until reaching 80% confluence. As previously reported [51], following trypsin dissociation, cells were stained with the following antibodies (mAbs): Rabbit-Thy-1 (CD90) and Rabbit-Endoglin (CD105) (Millipore, CA, USA), Mouse-SSEA4 (Cell Signaling Technology, MA, USA), Goat-Integrin β1 (CD29), Rabbit-HCAM (CD44) (Santa Cruz Biotechnology, CA, USA), Mouse-5’-Nucleotidase (CD73) (Gene Tex, CA, USA).

The expression of surface markers was analysed by indirect staining using secondary fluorochrome Alexa 488-conjugated antibodies (Abcam, Cambridge, UK). Non-specific fluorescence was assessed by using the secondary antibody alone. A minimum of 5,000 cells per sample was acquired and analysed using FACScan flow cytometer and Lysis II software (both from Becton Dickinson, San Jose, CA, USA).

The same cell samples were analyzed for nuclear stem cell markers, such as Goat- Sox2 (Santa Cruz Biotechnology, CA, USA) and Rabbit-Nanog and Rabbiti-Oct4, (Cell Signaling, MA, USA), after Fixation/Permeabilization process performed with the Staining Buffer Set (Miltenyi Biotec Inc, Auburn, CA, USA) optimized for nuclear staining.

After exposure to adipogenic differentiation medium, nuclear differentiation marker rabbit anti-PPAR (Santa Cruz, CA, USA) was analysed by using the Staining Buffer Set (Miltenyi Biotec Inc, Auburn, CA, USA).

### Cell treatment and preparation of cell extracts

Cells were treated with 10 µM Mevinolin (2-Methyl-1,2,3,7,8,8a-hexahydro-3,7-dimethyl-8-[2-(tetrahydro-4-hydroxy-6-oxo-2H-pyran-2-yl)ethyl]-1-naphthalenyl ester butanoic acid) for 16 hours (Sigma-Aldrich, St Louis, MO, USA), or with 50 µM Anacardic Acid (2-Hydroxy-6-pentadecylbenzoic acid) for 24 hours (Sigma-Aldrich, St Louis, MO, USA), or with 10 µM MG-132 (benzyloxycarbonyl-Leu-Leu-Leu-aldehyde) for 8 hours (Selleckchem, Houston, TX, USA).

Cell extracts were obtained as described by Maraldi et al. [27]. Briefly, subconfluent cells were extracted by addition of AT lysis buffer (20 mM Tris-Cl, pH 7.0; 1% Nonidet P-40; 150 mM NaCl; 10% glycerol; 10 mM EDTA; 20 mM NaF; 5 mM sodium pyrophosphate; and 1 mM Na_3_VO_4_) and freshly added Sigma Aldrich Protease Inhibitor Cocktail at 4°C for 30 min. Lysates were sonicated, cleared by centrifugation and immediately boiled in SDS sample buffer or used for immunoprecipitation experiments, as described below.

### Nuclei purification

Human AFSC nuclei were purified as reported by Guida et al. [52]. Briefly, 400 μl of nuclear isolation buffer (10 mM Tris-HCl, pH 7.8, 1% Nonidet P-40, 10 mM β-mercaptoethanol, 0.5 mM phenylmethylsulfonyl fluoride, 1 μg/ml aprotinin and leupeptin, and 5 mM NaF) were added to 5x10^6^ cells for 8 min on ice. MilliQ water (400 μl) was then added to swell cells for 3 min. Cells were sheared by passages through a 22-gauge needle. Nuclei were recovered by centrifugation at 400 × *g* at 4°C for 6 min and washed once in 400 μl of washing buffer (10 mM Tris-HCl, pH 7.4, and 2 mM MgCl_2_, plus inhibitors, as described earlier in the text). Supernatants (containing the cytosolic fractions) were further centrifuged for 30 min at 4000 × *g*. Isolated nuclear and cytoplasmic extracts were finally lysed in AT lysis buffer, sonicated, and cleared by centrifugation.

### Immunoprecipitation and Western blotting

Immunoprecipitation was performed as reported by Guida et al. [52]. Equal amounts of precleared lysates, whose protein concentration was determined by the Bradford method, were incubated overnight with Rabbit-Nox4 (Santa Cruz Biotechnology, CA, USA), Rabbit-Prelamin A (Diatheva, Fano, PU, Italy), Rabbit- Farnesylated Prelamin A (Diatheva, Fano, PU, Italy) (3 μg all). Then the two samples were treated with 30 μL of 50% (v/v) of protein A/G agarose slurry (GE Healthcare Bio-sciences, Uppsala, Sweden) at 4 °C with gentle rocking for 1 h. Pellets were washed twice with 20 mM Tris-Cl, pH 7.0; 1% Nonidet P-40; 150 mM NaCl; 10% glycerol; 10 mM EDTA; 20 mM NaF; 5 mM sodium pyrophosphate, once with 10 mM Tris-Cl, pH 7.4, boiled in SDS sample buffer, and centrifuged. Supernatants were loaded onto SDS-polyacrylamide gel, blotted on Immobilon-P membranes (Millipore, Waltham, MA, USA), and processed by Western blot with the indicated antibodies, detected by Supersignal substrate chemiluminescence detection kit (Pierce, Rockford, IL, USA). Quantitation of the signal was obtained by chemiluminescence detection on a Kodak Image Station 440CF and analysis with the Kodak 1D Image software. Primary antibodies were raised against the following molecules: Rabbit-p16 (Abcam, Cambridge, UK), Mouse-Tubulin (Sigma-Aldrich, St Louis, MO, USA), Mouse-SIRT1 (Cell Signaling Technology, MA, USA), Rabbit-Nox4 (Santa Cruz Biotechnology, CA, USA), Rabbit-Prelamin A (Diatheva, Fano, PU, Italy), Mouse-Lamin A/C (Santa Cruz Biotechnology, CA, USA), Rabbit-SUMO1 (Cell Signaling Technology, MA, USA).

### SDS-PAGE and Coomassie brilliant blue staining

Nox4- immunoprecipitated samples were separated by 10% SDS-PAGE and gels were then stained in the Coomassie brilliant blue solution (0.1% Coomassie blue in 10% acetic acid, 45% methanol) and shaken at room temperature for 1 h. The gels were destained by soaking for 2 h in destaining solution (10% acetic acid, 30% methanol).

### Mass spectrometry

In-gel trypsin digestion and mass spectrometry analyses were performed as discussed in Shevchenko et al. [53]. Briefly, the stained bands were reduced in gel pieces that were treated with solution A (1:1 mixture of acetonitrile: 100 mM ammonium bicarbonate) for 30 min. The gel slices were subjected to reduction of disulfide bonds by 10 mM DTT at 56°C for 30 min. Alkylation step was then performed with 55 mM iodoacetamide for 20 min at room temperature in dark. Before trypsin digestion, the rehydration and dehydration steps were again performed with ammonium bicarbonate and solution A. Then samples were incubated at RT with acetonitrile until gel pieces become white. Trypsin digestion was performed by incubating the dry gel slices overnight in 1 µg of trypsin in ammonium bicarbonate (Trypsin Sigma) at 37°C. Following digestion, the tryptic digested fragments present in the supernatant were collected and lyophilized. Before mass spectrometry analysis, the lyophilized powder was dissolved in 0.1% TFA in 50% acetonitrile. The expressed proteins were identified using LC/MS/MS 6410B mass spectrometer. Protein identification against the peak list was performed in MASCOT version 2.1 with Swiss-Prot and cRAP database as the search engine. The search parameters for database search using Mascot were given as, taxonomy: human; enzyme used for digestion: trypsin with one missed cleavage allowed; fixed modification specified as carbamidomethylation (C), and oxidation (M) and deamidated (NQ) as variable modifications. The peptide mass tolerance was set as 40 ppm and 0.08 Da for MS/MS tolerance.

### Immunofluorescence and confocal microscopy

For immunofluorescence analysis, samples were processed as previously described [52]. Confocal imaging was performed by a Nikon A1 confocal laser scanning microscope, as previously described [54]. Primary antibodies were raised against the following molecules: Rabbit-p53 (Flarebio, MD, USA), Mouse-p21 (Novus Biologicals, CO, USA), Mouse-pH2A (Millipore, CA, USA), Rabbit-Nox4 (Santa Cruz Biotechnology, CA, USA), Mouse-Nox4 (Novus Biologicals, CO, USA), Rabbit-Prelamin A (Diatheva, Fano, PU, Italy), Rabbit-Farnesylated Prelamin A (Diatheva, Fano, PU, Italy), Mouse-PML (Santa Cruz Biotechnology, CA, USA), Mouse-SUMO1 (Novus Biologicals, CO, USA).

The confocal serial sections were processed with ImageJ software to obtain three-dimensional projections. The image rendering was performed by Adobe Photoshop software.

### Statistical analysis

*In vitro* experiments were performed in triplicate. For quantitative comparisons, values were reported as mean ± SD based on triplicate analysis for each sample. To test the significance of observed differences among the study groups Student’s t-test or One-way Anova with Bonferroni post hoc test were applied. A P value <0.05 was considered to be statistically significant. Statistical analysis and plot layout were obtained by using GraphPad Prism® release 5.0 software.

### Ethics approval and consent to participate

An informed consent allowing the use of clinical data and biological samples for the specified research purpose (protocol 2015/0004362 of 02.24.2015) was signed by all infertile couples before treatment and collected by the Unit of Obstetrics & Gynecology, IRCCS - ASMN of Reggio Emilia (Italy).

## SUPPLEMENTARY MATERIAL

Supplementary Figure S1

Supplementary Figure S2

Supplementary Figure S3

Supplementary Figure S4

Supplementary Figure S5

Supplementary Figure S6
