# Supplementary material for: Nuclear Nox4 interaction with prelamin A is associated with nuclear redox control of stem cell aging
Source: Aging (Albany NY). 2018 Oct 24;10(10):2911–34. doi: 10.18632/aging.101599 (PMC6224265; doi:10.18632/aging.101599)
Supplement: Supplementary Figure S1 [file aging-10-101599-s001.pdf]

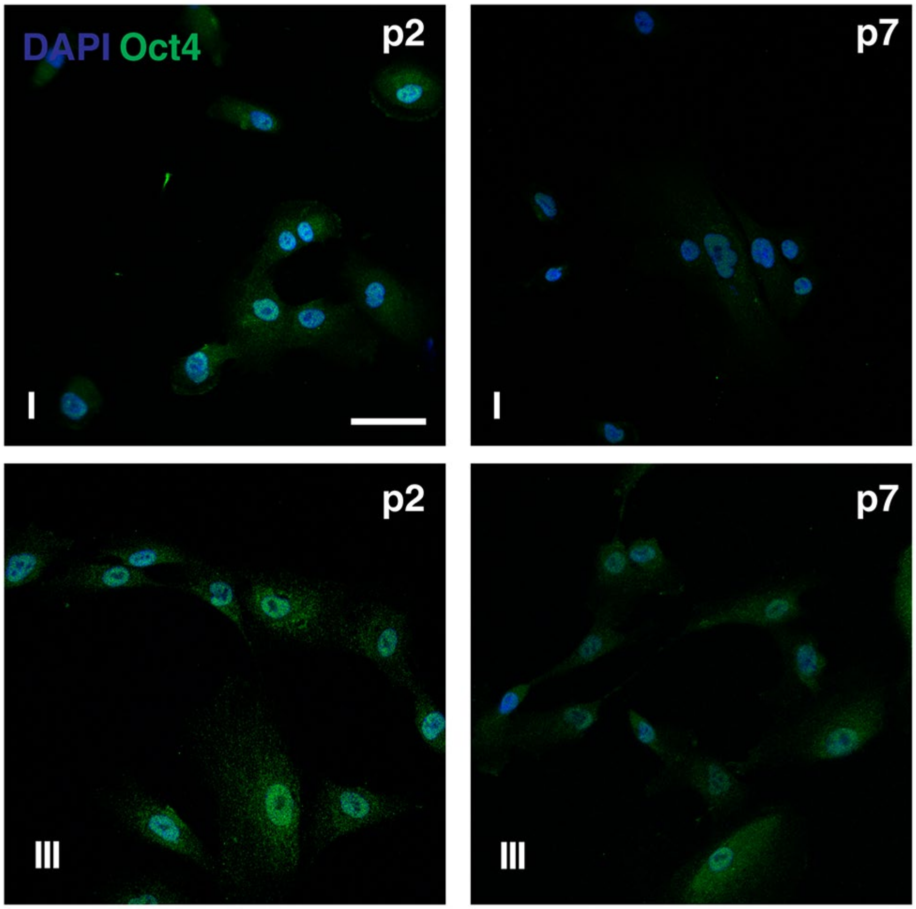

Supplementary Figure S1. Oct4 decrease during culture passages. Representative images of AFSC group I and III, at passage 2 and 7, labeled with DAPI (blue), Oct4 (green). Scale bar=10 µm.
