# Supplementary material for: Nuclear Nox4 interaction with prelamin A is associated with nuclear redox control of stem cell aging
Source: Aging (Albany NY). 2018 Oct 24;10(10):2911–34. doi: 10.18632/aging.101599 (PMC6224265; doi:10.18632/aging.101599)
Supplement: Supplementary Figure S2 [file aging-10-101599-s002.pdf]

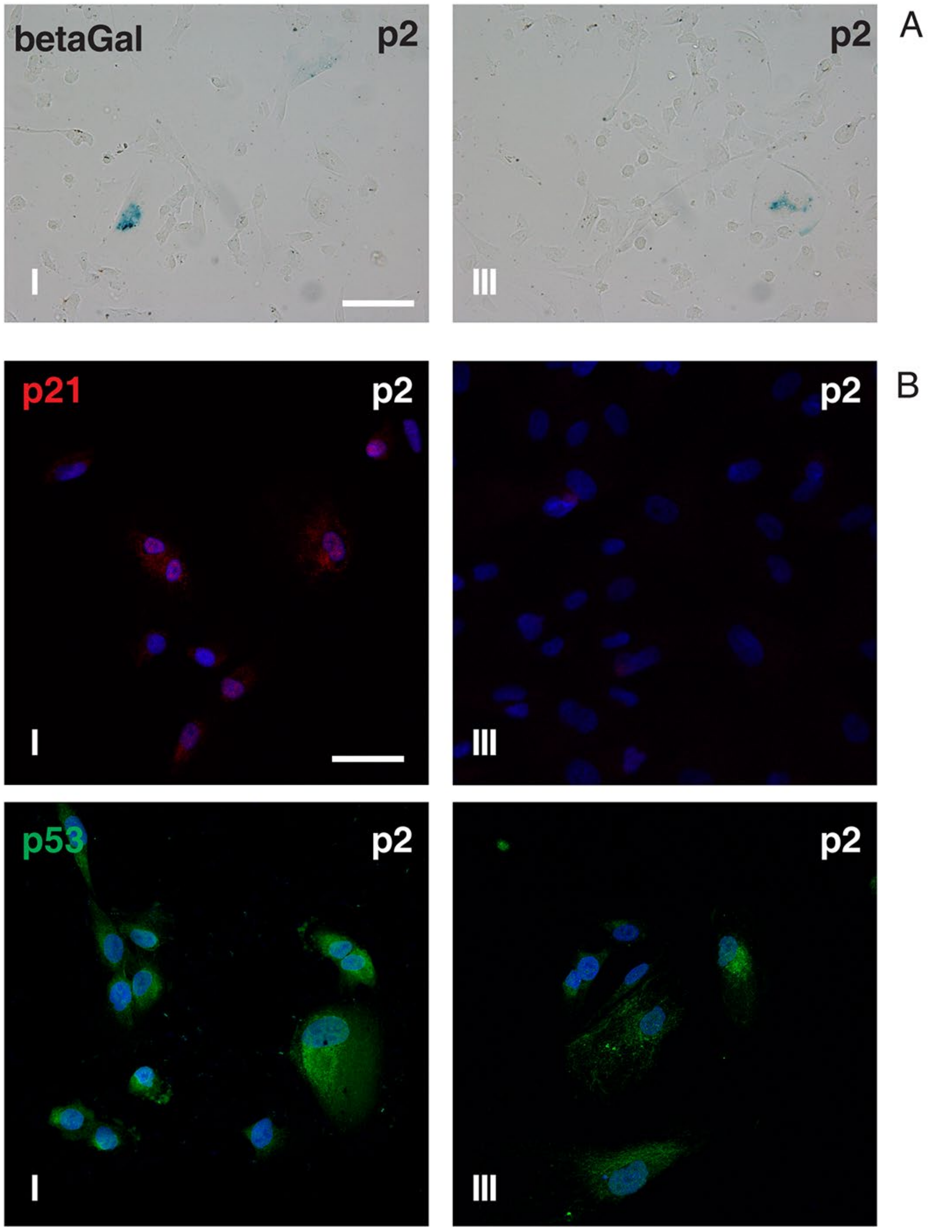

Supplementary Figure S2. Senescence marker at early passages for AFSC groups. (**A**) Representative images of beta-galactosidase assay performed in group I and III samples at passage 2. Senescent cells are blue stained. Scale bar=30 µm. (**B**) Representative images of AFSC group II labeled with DAPI (blue), and p21(red) or p53 (green). Scale bar=10 µm.
