# Supplementary material for: Nuclear Nox4 interaction with prelamin A is associated with nuclear redox control of stem cell aging
Source: Aging (Albany NY). 2018 Oct 24;10(10):2911–34. doi: 10.18632/aging.101599 (PMC6224265; doi:10.18632/aging.101599)
Supplement: Supplementary Figure S3 [file aging-10-101599-s003.pdf]

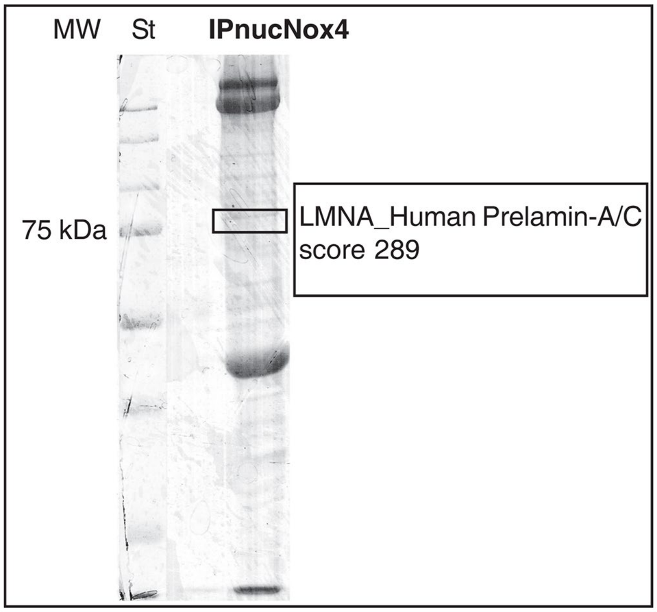

Supplementary Figure S3. Mass spectrometry experiment. Nuclear lysate of AFSC of group II was subjected to immunoprecipitation with Nox4 antibody. Image of Coomassie staining of proteins in IPnucNox4 separated by SDS-PAGE is shown. The mass spectrometry revealed that prelamin A/C protein was present, beside others, in the band highlighted in black.
