# Supplementary material for: Nuclear Nox4 interaction with prelamin A is associated with nuclear redox control of stem cell aging
Source: Aging (Albany NY). 2018 Oct 24;10(10):2911–34. doi: 10.18632/aging.101599 (PMC6224265; doi:10.18632/aging.101599)
Supplement: Supplementary Figure S4 [file aging-10-101599-s004.pdf]

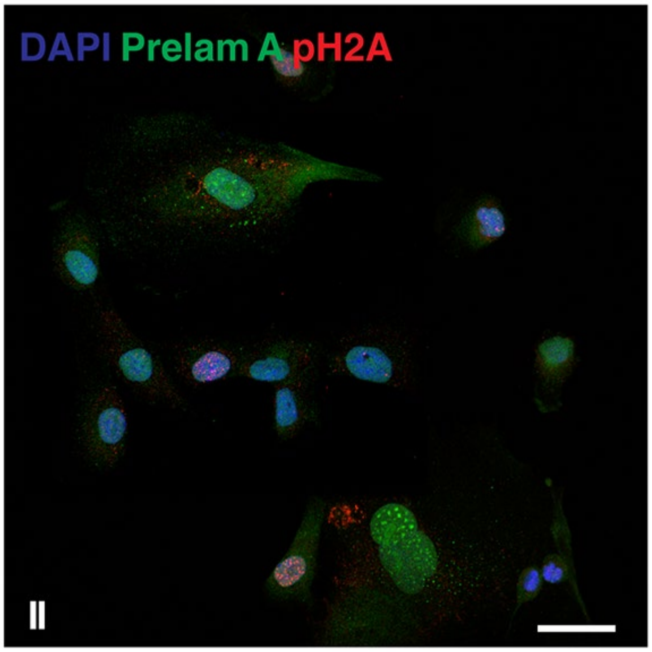

Supplementary Figure S4. Prelamin A accumulation and DNA damage marker. Representative images of AFSC group II labelled with DAPI (blue), Prelamin A (green) and pH2A (red). Scale bar=10 µm.
