# Supplementary material for: Nuclear Nox4 interaction with prelamin A is associated with nuclear redox control of stem cell aging
Source: Aging (Albany NY). 2018 Oct 24;10(10):2911–34. doi: 10.18632/aging.101599 (PMC6224265; doi:10.18632/aging.101599)
Supplement: Supplementary Figure S5 [file aging-10-101599-s005.pdf]

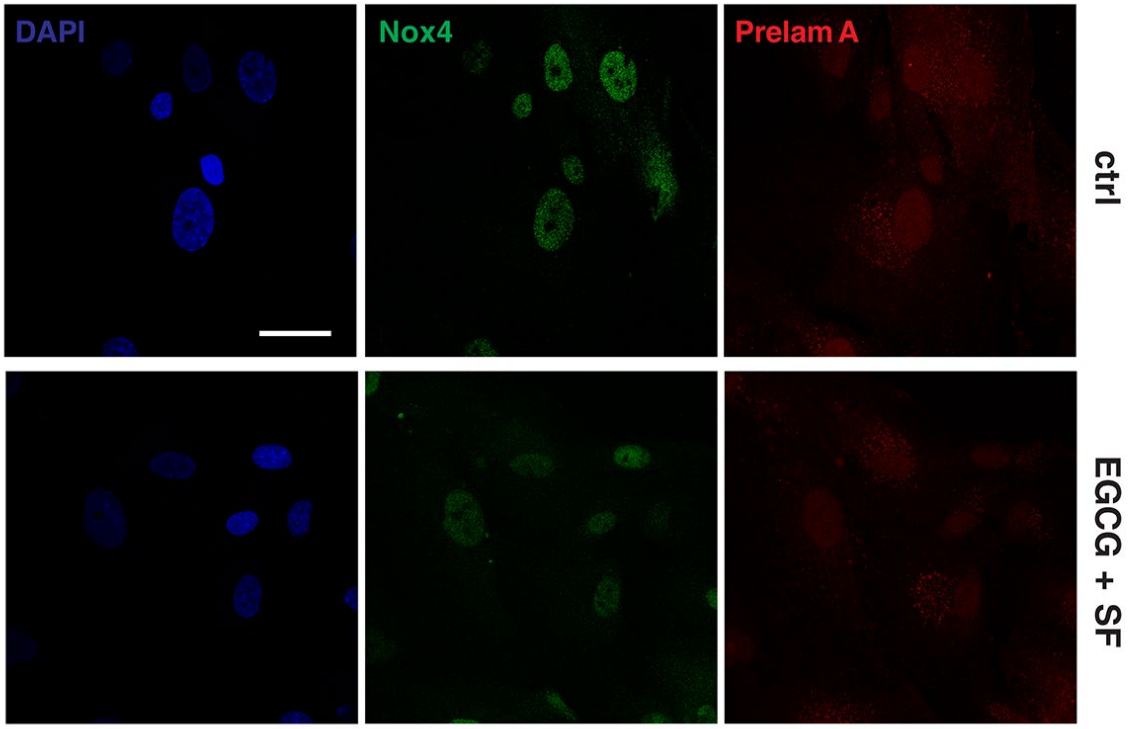

Supplementary Figure S5. Effect of ROS decrease on Nox4 and prelamin A accumulation. Representative images of AFSC group I and III, treated or not with 1 µM sulforaphane (SF) and 10 µM epigallocathechin gallate (EGCG) for 11 days, labelled with DAPI (blue), Nox4 (green) and Prelamin A (red). Scale bar=10 µm.
