# Supplementary material for: Nuclear Nox4 interaction with prelamin A is associated with nuclear redox control of stem cell aging
Source: Aging (Albany NY). 2018 Oct 24;10(10):2911–34. doi: 10.18632/aging.101599 (PMC6224265; doi:10.18632/aging.101599)
Supplement: Supplementary Figure S6 [file aging-10-101599-s006.pdf]

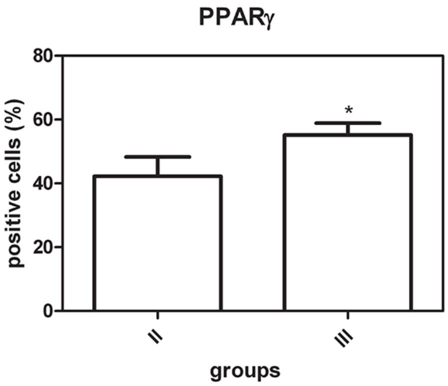

Supplementary Figure S6. Adipogenic differentiation capability. Graph showing the cytofluorimetric analysis of PPARγ expression among the groups II and III, after exposure to adipogenic differentiation. Four samples were analysed for each group. *P < 0.05 = significantly different from group II.
